# Supplementary material for: Modeling glioblastoma heterogeneity as a dynamic network of cell states
Source: Mol Syst Biol. 2021 Sep 16;17(9):e10105. doi: 10.15252/msb.202010105 (PMC8444284; doi:10.15252/msb.202010105)
Supplement: Supplementary file 6 — Source Data for Figure 5 [file MSB-17-e10105-s004.zip › Figure5A_sourcedata/GSEA_3017/nosigdb_stateF.GseaPreranked.1622795015644/GAROFANO_IAVARONE_PPR_SUBTYPE.html]

Details for gene set GAROFANO\_IAVARONE\_PPR\_SUBTYPE[GSEA]

|  || Dataset | state33017 |
| Phenotype | NoPhenotypeAvailable |
| Upregulated in class | na\_neg |
| GeneSet | GAROFANO\_IAVARONE\_PPR\_SUBTYPE |
| Enrichment Score (ES) | -0.75611806 |
| Normalized Enrichment Score (NES) | -5.787385 |
| Nominal p-value | 0.0 |
| FDR q-value | 0.0 |
| FWER p-Value | 0.0 |
Table: GSEA Results Summary

  

Fig 1: Enrichment plot: GAROFANO\_IAVARONE\_PPR\_SUBTYPE      
 Profile of the Running ES Score & Positions of GeneSet Members on the Rank Ordered List

  

| PROBE | GENE SYMBOL | GENE\_TITLE | RANK IN GENE LIST | RANK METRIC SCORE | RUNNING ES | CORE ENRICHMENT || 1 | MAP2 |  |  | 455 | 0.448 | -0.2234 | No |
| 2 | C2orf80 |  |  | 486 | 0.439 | -0.2356 | No |
| 3 | NTRK3 |  |  | 621 | 0.391 | -0.2998 | No |
| 4 | KCNK10 |  |  | 697 | 0.372 | -0.3347 | No |
| 5 | GTF2IRD2 |  |  | 809 | 0.345 | -0.3877 | No |
| 6 | PGAP1 |  |  | 823 | 0.342 | -0.3920 | No |
| 7 | ASB3 |  |  | 851 | 0.336 | -0.4034 | No |
| 8 | GAB1 |  |  | 958 | 0.316 | -0.4541 | No |
| 9 | MEX3A |  |  | 1474 | -0.251 | -0.7086 | No |
| 10 | DNA2 |  |  | 1479 | -0.252 | -0.7090 | No |
| 11 | SOX11 |  |  | 1492 | -0.256 | -0.7133 | No |
| 12 | DCLRE1C |  |  | 1493 | -0.257 | -0.7117 | No |
| 13 | PKNOX1 |  |  | 1498 | -0.258 | -0.7121 | No |
| 14 | HNRNPA2B1 |  |  | 1503 | -0.261 | -0.7124 | No |
| 15 | BLM |  |  | 1507 | -0.262 | -0.7123 | No |
| 16 | HCFC1 |  |  | 1515 | -0.264 | -0.7141 | No |
| 17 | MSH6 |  |  | 1517 | -0.264 | -0.7129 | No |
| 18 | NIF3L1 |  |  | 1519 | -0.265 | -0.7118 | No |
| 19 | CEP78 |  |  | 1524 | -0.265 | -0.7121 | No |
| 20 | CBX2 |  |  | 1526 | -0.265 | -0.7109 | No |
| 21 | NSMCE4A |  |  | 1533 | -0.267 | -0.7122 | No |
| 22 | ZNF100 |  |  | 1536 | -0.268 | -0.7115 | No |
| 23 | MAD2L2 |  |  | 1539 | -0.269 | -0.7108 | No |
| 24 | CCDC138 |  |  | 1541 | -0.270 | -0.7096 | No |
| 25 | ZNF43 |  |  | 1544 | -0.270 | -0.7089 | No |
| 26 | SAFB |  |  | 1549 | -0.271 | -0.7092 | No |
| 27 | FAM76B |  |  | 1552 | -0.272 | -0.7085 | No |
| 28 | CREB5 |  |  | 1561 | -0.275 | -0.7107 | No |
| 29 | KHDRBS1 |  |  | 1573 | -0.278 | -0.7144 | No |
| 30 | ZNF704 |  |  | 1588 | -0.284 | -0.7196 | No |
| 31 | RCC1 |  |  | 1604 | -0.286 | -0.7253 | No |
| 32 | CCDC150 |  |  | 1605 | -0.286 | -0.7235 | No |
| 33 | POLA2 |  |  | 1622 | -0.291 | -0.7296 | No |
| 34 | TMEM97 |  |  | 1629 | -0.293 | -0.7307 | No |
| 35 | GINS1 |  |  | 1634 | -0.295 | -0.7309 | No |
| 36 | SNRPA |  |  | 1637 | -0.296 | -0.7300 | No |
| 37 | SUV39H2 |  |  | 1643 | -0.298 | -0.7306 | No |
| 38 | KLHL23 |  |  | 1674 | -0.308 | -0.7436 | No |
| 39 | OIP5 |  |  | 1686 | -0.314 | -0.7471 | No |
| 40 | TNFAIP8L1 |  |  | 1695 | -0.317 | -0.7491 | No |
| 41 | ZNF93 |  |  | 1699 | -0.318 | -0.7486 | No |
| 42 | PRIM1 |  |  | 1703 | -0.320 | -0.7480 | No |
| 43 | HAUS1 |  |  | 1708 | -0.322 | -0.7480 | No |
| 44 | TRIM28 |  |  | 1709 | -0.322 | -0.7460 | No |
| 45 | PARP1 |  |  | 1713 | -0.323 | -0.7454 | No |
| 46 | SASS6 |  |  | 1719 | -0.325 | -0.7459 | No |
| 47 | RAD51 |  |  | 1723 | -0.326 | -0.7453 | No |
| 48 | CDC25C |  |  | 1724 | -0.327 | -0.7433 | No |
| 49 | BRD7 |  |  | 1730 | -0.330 | -0.7437 | No |
| 50 | VEZF1 |  |  | 1732 | -0.331 | -0.7421 | No |
| 51 | NEIL3 |  |  | 1733 | -0.331 | -0.7400 | No |
| 52 | PLK4 |  |  | 1740 | -0.336 | -0.7409 | No |
| 53 | RMI1 |  |  | 1743 | -0.337 | -0.7397 | No |
| 54 | STIL |  |  | 1744 | -0.337 | -0.7376 | No |
| 55 | EXOSC9 |  |  | 1746 | -0.338 | -0.7360 | No |
| 56 | PTBP1 |  |  | 1756 | -0.342 | -0.7383 | No |
| 57 | ZNF90 |  |  | 1763 | -0.345 | -0.7391 | No |
| 58 | WDR76 |  |  | 1783 | -0.355 | -0.7463 | No |
| 59 | BRIP1 |  |  | 1790 | -0.357 | -0.7471 | No |
| 60 | POC1A |  |  | 1794 | -0.360 | -0.7463 | No |
| 61 | LIG1 |  |  | 1797 | -0.361 | -0.7450 | No |
| 62 | KDM1A |  |  | 1814 | -0.371 | -0.7506 | No |
| 63 | SAP30 |  |  | 1826 | -0.378 | -0.7537 | Yes |
| 64 | C18orf54 |  |  | 1830 | -0.380 | -0.7528 | Yes |
| 65 | CDC45 |  |  | 1836 | -0.382 | -0.7529 | Yes |
| 66 | BARD1 |  |  | 1839 | -0.384 | -0.7515 | Yes |
| 67 | ZNF519 |  |  | 1841 | -0.385 | -0.7496 | Yes |
| 68 | MASTL |  |  | 1843 | -0.385 | -0.7477 | Yes |
| 69 | FANCA |  |  | 1844 | -0.385 | -0.7452 | Yes |
| 70 | CEP135 |  |  | 1855 | -0.393 | -0.7477 | Yes |
| 71 | PLEKHO1 |  |  | 1858 | -0.393 | -0.7463 | Yes |
| 72 | KIF18B |  |  | 1860 | -0.393 | -0.7443 | Yes |
| 73 | WDR62 |  |  | 1861 | -0.396 | -0.7418 | Yes |
| 74 | FANCB |  |  | 1862 | -0.396 | -0.7393 | Yes |
| 75 | E2F8 |  |  | 1864 | -0.396 | -0.7373 | Yes |
| 76 | EXOSC8 |  |  | 1865 | -0.397 | -0.7348 | Yes |
| 77 | PKMYT1 |  |  | 1876 | -0.402 | -0.7372 | Yes |
| 78 | CENPO |  |  | 1884 | -0.410 | -0.7381 | Yes |
| 79 | RBMX |  |  | 1885 | -0.412 | -0.7356 | Yes |
| 80 | HNRNPA0 |  |  | 1889 | -0.415 | -0.7344 | Yes |
| 81 | POLQ |  |  | 1891 | -0.417 | -0.7323 | Yes |
| 82 | SAE1 |  |  | 1893 | -0.417 | -0.7302 | Yes |
| 83 | ASF1B |  |  | 1897 | -0.419 | -0.7290 | Yes |
| 84 | DPF1 |  |  | 1909 | -0.429 | -0.7318 | Yes |
| 85 | BAZ1B |  |  | 1910 | -0.429 | -0.7291 | Yes |
| 86 | MNS1 |  |  | 1913 | -0.430 | -0.7274 | Yes |
| 87 | POLD3 |  |  | 1918 | -0.436 | -0.7266 | Yes |
| 88 | GINS2 |  |  | 1932 | -0.445 | -0.7303 | Yes |
| 89 | CCNF |  |  | 1947 | -0.451 | -0.7344 | Yes |
| 90 | DDX11 |  |  | 1952 | -0.456 | -0.7335 | Yes |
| 91 | RFC2 |  |  | 1954 | -0.457 | -0.7312 | Yes |
| 92 | ZNF22 |  |  | 1955 | -0.457 | -0.7283 | Yes |
| 93 | CDT1 |  |  | 1961 | -0.460 | -0.7279 | Yes |
| 94 | ZWILCH |  |  | 1962 | -0.461 | -0.7250 | Yes |
| 95 | SEPHS1 |  |  | 1967 | -0.464 | -0.7240 | Yes |
| 96 | DNMT1 |  |  | 1969 | -0.465 | -0.7216 | Yes |
| 97 | POP7 |  |  | 1981 | -0.474 | -0.7241 | Yes |
| 98 | NASP |  |  | 1988 | -0.481 | -0.7241 | Yes |
| 99 | TIMELESS |  |  | 1989 | -0.482 | -0.7210 | Yes |
| 100 | CDC6 |  |  | 1991 | -0.483 | -0.7185 | Yes |
| 101 | MCM4 |  |  | 1993 | -0.487 | -0.7159 | Yes |
| 102 | SKA1 |  |  | 1996 | -0.490 | -0.7138 | Yes |
| 103 | SPAG5 |  |  | 1997 | -0.490 | -0.7107 | Yes |
| 104 | TK1 |  |  | 2003 | -0.497 | -0.7101 | Yes |
| 105 | CTCF |  |  | 2010 | -0.502 | -0.7099 | Yes |
| 106 | CHEK1 |  |  | 2013 | -0.504 | -0.7077 | Yes |
| 107 | HIRIP3 |  |  | 2017 | -0.508 | -0.7060 | Yes |
| 108 | FEN1 |  |  | 2018 | -0.511 | -0.7028 | Yes |
| 109 | MAZ |  |  | 2019 | -0.511 | -0.6996 | Yes |
| 110 | SMC1A |  |  | 2021 | -0.513 | -0.6969 | Yes |
| 111 | RRM1 |  |  | 2023 | -0.516 | -0.6941 | Yes |
| 112 | RANBP1 |  |  | 2024 | -0.517 | -0.6909 | Yes |
| 113 | SRRT |  |  | 2025 | -0.517 | -0.6876 | Yes |
| 114 | HAUS8 |  |  | 2026 | -0.517 | -0.6844 | Yes |
| 115 | DEPDC1B |  |  | 2037 | -0.527 | -0.6860 | Yes |
| 116 | HELLS |  |  | 2038 | -0.528 | -0.6827 | Yes |
| 117 | TRIP13 |  |  | 2044 | -0.530 | -0.6818 | Yes |
| 118 | NCL |  |  | 2045 | -0.532 | -0.6785 | Yes |
| 119 | TROAP |  |  | 2046 | -0.533 | -0.6751 | Yes |
| 120 | HNRNPM |  |  | 2048 | -0.533 | -0.6723 | Yes |
| 121 | NCAPD3 |  |  | 2051 | -0.537 | -0.6699 | Yes |
| 122 | ILF3 |  |  | 2052 | -0.537 | -0.6665 | Yes |
| 123 | KIF22 |  |  | 2053 | -0.541 | -0.6631 | Yes |
| 124 | CENPJ |  |  | 2054 | -0.544 | -0.6597 | Yes |
| 125 | CKLF |  |  | 2055 | -0.544 | -0.6563 | Yes |
| 126 | INCENP |  |  | 2061 | -0.556 | -0.6553 | Yes |
| 127 | STMN1 |  |  | 2063 | -0.567 | -0.6522 | Yes |
| 128 | PTMA |  |  | 2065 | -0.570 | -0.6491 | Yes |
| 129 | DTL |  |  | 2066 | -0.573 | -0.6455 | Yes |
| 130 | FANCD2 |  |  | 2068 | -0.577 | -0.6424 | Yes |
| 131 | ZWINT |  |  | 2069 | -0.577 | -0.6387 | Yes |
| 132 | CEP152 |  |  | 2070 | -0.578 | -0.6351 | Yes |
| 133 | RTKN2 |  |  | 2071 | -0.579 | -0.6314 | Yes |
| 134 | RACGAP1 |  |  | 2072 | -0.579 | -0.6278 | Yes |
| 135 | CENPA |  |  | 2073 | -0.586 | -0.6241 | Yes |
| 136 | MND1 |  |  | 2074 | -0.586 | -0.6204 | Yes |
| 137 | LSM4 |  |  | 2076 | -0.587 | -0.6172 | Yes |
| 138 | SKA3 |  |  | 2077 | -0.590 | -0.6135 | Yes |
| 139 | AURKB |  |  | 2079 | -0.598 | -0.6102 | Yes |
| 140 | CENPK |  |  | 2082 | -0.607 | -0.6074 | Yes |
| 141 | HNRNPR |  |  | 2083 | -0.612 | -0.6036 | Yes |
| 142 | CKAP5 |  |  | 2086 | -0.623 | -0.6006 | Yes |
| 143 | PLK1 |  |  | 2087 | -0.624 | -0.5967 | Yes |
| 144 | PIF1 |  |  | 2088 | -0.627 | -0.5928 | Yes |
| 145 | HNRNPU |  |  | 2090 | -0.628 | -0.5893 | Yes |
| 146 | SSRP1 |  |  | 2091 | -0.630 | -0.5853 | Yes |
| 147 | KIF18A |  |  | 2093 | -0.636 | -0.5818 | Yes |
| 148 | FAM111B |  |  | 2094 | -0.637 | -0.5778 | Yes |
| 149 | DNAJC9 |  |  | 2097 | -0.641 | -0.5748 | Yes |
| 150 | RFC3 |  |  | 2098 | -0.642 | -0.5708 | Yes |
| 151 | BRCA2 |  |  | 2100 | -0.650 | -0.5672 | Yes |
| 152 | NCAPH |  |  | 2104 | -0.652 | -0.5645 | Yes |
| 153 | CKAP2L |  |  | 2109 | -0.663 | -0.5624 | Yes |
| 154 | MRTO4 |  |  | 2113 | -0.683 | -0.5596 | Yes |
| 155 | CDCA2 |  |  | 2120 | -0.705 | -0.5581 | Yes |
| 156 | CENPM |  |  | 2125 | -0.709 | -0.5556 | Yes |
| 157 | HNRNPD |  |  | 2126 | -0.713 | -0.5511 | Yes |
| 158 | RAD51AP1 |  |  | 2127 | -0.715 | -0.5466 | Yes |
| 159 | CDCA8 |  |  | 2129 | -0.722 | -0.5426 | Yes |
| 160 | HNRNPAB |  |  | 2130 | -0.729 | -0.5380 | Yes |
| 161 | CKAP2 |  |  | 2131 | -0.729 | -0.5334 | Yes |
| 162 | ATAD5 |  |  | 2133 | -0.742 | -0.5292 | Yes |
| 163 | KIF2C |  |  | 2136 | -0.756 | -0.5255 | Yes |
| 164 | BRCA1 |  |  | 2138 | -0.758 | -0.5212 | Yes |
| 165 | KIF14 |  |  | 2141 | -0.773 | -0.5173 | Yes |
| 166 | NDC80 |  |  | 2142 | -0.775 | -0.5125 | Yes |
| 167 | H2AFV |  |  | 2143 | -0.779 | -0.5076 | Yes |
| 168 | KIF23 |  |  | 2145 | -0.782 | -0.5031 | Yes |
| 169 | GMNN |  |  | 2146 | -0.785 | -0.4982 | Yes |
| 170 | LMNB2 |  |  | 2147 | -0.786 | -0.4932 | Yes |
| 171 | MELK |  |  | 2148 | -0.786 | -0.4883 | Yes |
| 172 | SMC3 |  |  | 2150 | -0.800 | -0.4838 | Yes |
| 173 | KIFC1 |  |  | 2151 | -0.802 | -0.4787 | Yes |
| 174 | DUT |  |  | 2155 | -0.824 | -0.4750 | Yes |
| 175 | NCAPG2 |  |  | 2156 | -0.827 | -0.4698 | Yes |
| 176 | NUCKS1 |  |  | 2158 | -0.832 | -0.4651 | Yes |
| 177 | LSM5 |  |  | 2159 | -0.835 | -0.4598 | Yes |
| 178 | MCM7 |  |  | 2160 | -0.839 | -0.4545 | Yes |
| 179 | ECT2 |  |  | 2163 | -0.846 | -0.4502 | Yes |
| 180 | HMGB3 |  |  | 2164 | -0.852 | -0.4448 | Yes |
| 181 | SPC24 |  |  | 2166 | -0.853 | -0.4400 | Yes |
| 182 | ARHGAP11A |  |  | 2167 | -0.855 | -0.4346 | Yes |
| 183 | DTYMK |  |  | 2168 | -0.858 | -0.4292 | Yes |
| 184 | MCM10 |  |  | 2169 | -0.858 | -0.4238 | Yes |
| 185 | BUB1 |  |  | 2170 | -0.860 | -0.4184 | Yes |
| 186 | FOXM1 |  |  | 2171 | -0.861 | -0.4130 | Yes |
| 187 | CDCA5 |  |  | 2172 | -0.866 | -0.4075 | Yes |
| 188 | LBR |  |  | 2173 | -0.870 | -0.4020 | Yes |
| 189 | BUB1B |  |  | 2174 | -0.871 | -0.3966 | Yes |
| 190 | NUF2 |  |  | 2176 | -0.877 | -0.3915 | Yes |
| 191 | KPNA2 |  |  | 2178 | -0.894 | -0.3864 | Yes |
| 192 | CCNA2 |  |  | 2181 | -0.902 | -0.3817 | Yes |
| 193 | CHAF1A |  |  | 2182 | -0.903 | -0.3761 | Yes |
| 194 | KIF15 |  |  | 2185 | -0.915 | -0.3713 | Yes |
| 195 | KIF4A |  |  | 2186 | -0.921 | -0.3655 | Yes |
| 196 | CENPH |  |  | 2191 | -0.943 | -0.3615 | Yes |
| 197 | CENPW |  |  | 2192 | -0.943 | -0.3556 | Yes |
| 198 | TUBA1B |  |  | 2193 | -0.947 | -0.3497 | Yes |
| 199 | LMNB1 |  |  | 2194 | -0.950 | -0.3437 | Yes |
| 200 | HJURP |  |  | 2195 | -0.962 | -0.3376 | Yes |
| 201 | UBE2T |  |  | 2196 | -0.965 | -0.3316 | Yes |
| 202 | FANCI |  |  | 2198 | -0.966 | -0.3260 | Yes |
| 203 | CCNB2 |  |  | 2200 | -0.983 | -0.3203 | Yes |
| 204 | CKS1B |  |  | 2201 | -0.987 | -0.3141 | Yes |
| 205 | ESCO2 |  |  | 2204 | -1.004 | -0.3087 | Yes |
| 206 | SMC2 |  |  | 2205 | -1.006 | -0.3024 | Yes |
| 207 | KIF11 |  |  | 2206 | -1.008 | -0.2961 | Yes |
| 208 | EZH2 |  |  | 2207 | -1.017 | -0.2897 | Yes |
| 209 | SPC25 |  |  | 2208 | -1.019 | -0.2832 | Yes |
| 210 | XRCC2 |  |  | 2210 | -1.028 | -0.2773 | Yes |
| 211 | CENPE |  |  | 2211 | -1.036 | -0.2707 | Yes |
| 212 | NUDT1 |  |  | 2214 | -1.054 | -0.2651 | Yes |
| 213 | MYBL2 |  |  | 2215 | -1.058 | -0.2584 | Yes |
| 214 | CLSPN |  |  | 2216 | -1.060 | -0.2518 | Yes |
| 215 | CENPN |  |  | 2217 | -1.079 | -0.2450 | Yes |
| 216 | UBE2C |  |  | 2218 | -1.081 | -0.2382 | Yes |
| 217 | H2AFZ |  |  | 2220 | -1.090 | -0.2318 | Yes |
| 218 | HMGB1 |  |  | 2221 | -1.116 | -0.2248 | Yes |
| 219 | NCAPG |  |  | 2222 | -1.122 | -0.2177 | Yes |
| 220 | H2AFX |  |  | 2223 | -1.146 | -0.2105 | Yes |
| 221 | CDK1 |  |  | 2225 | -1.153 | -0.2037 | Yes |
| 222 | ANP32E |  |  | 2227 | -1.200 | -0.1967 | Yes |
| 223 | MAD2L1 |  |  | 2228 | -1.204 | -0.1891 | Yes |
| 224 | UBE2S |  |  | 2229 | -1.205 | -0.1815 | Yes |
| 225 | GTSE1 |  |  | 2230 | -1.220 | -0.1739 | Yes |
| 226 | HIST2H2AC |  |  | 2231 | -1.231 | -0.1661 | Yes |
| 227 | DBF4 |  |  | 2232 | -1.247 | -0.1583 | Yes |
| 228 | HMGN2 |  |  | 2233 | -1.267 | -0.1503 | Yes |
| 229 | RRM2 |  |  | 2234 | -1.268 | -0.1423 | Yes |
| 230 | SMC4 |  |  | 2236 | -1.281 | -0.1347 | Yes |
| 231 | ATAD2 |  |  | 2238 | -1.381 | -0.1265 | Yes |
| 232 | TMPO |  |  | 2239 | -1.382 | -0.1178 | Yes |
| 233 | PRC1 |  |  | 2240 | -1.397 | -0.1090 | Yes |
| 234 | HMGB2 |  |  | 2241 | -1.469 | -0.0998 | Yes |
| 235 | BIRC5 |  |  | 2242 | -1.469 | -0.0906 | Yes |
| 236 | ASPM |  |  | 2244 | -1.490 | -0.0817 | Yes |
| 237 | TOP2A |  |  | 2246 | -1.562 | -0.0723 | Yes |
| 238 | NUSAP1 |  |  | 2247 | -1.600 | -0.0623 | Yes |
| 239 | TPX2 |  |  | 2248 | -1.609 | -0.0521 | Yes |
| 240 | CENPF |  |  | 2249 | -1.691 | -0.0415 | Yes |
| 241 | HIST1H1B |  |  | 2251 | -1.796 | -0.0307 | Yes |
| 242 | MKI67 |  |  | 2252 | -1.892 | -0.0188 | Yes |
| 243 | HIST1H4C |  |  | 2253 | -2.984 | -0.0000 | Yes |
Table: GSEA details [plain text format]

  

Fig 2: GAROFANO\_IAVARONE\_PPR\_SUBTYPE: Random ES distribution      
 Gene set null distribution of ES for **GAROFANO\_IAVARONE\_PPR\_SUBTYPE**
